# Sustainability of community-based women’s groups: reflections from a participatory intervention for newborn and maternal health in Nepal

**DOI:** 10.1093/cdj/bsy017

**Published:** 2018-04-30

**Authors:** Annemijn E C Sondaal, Kirti M Tumbahangphe, Rishi Neupane, Dharma S Manandhar, Anthony Costello, Joanna Morrison

## Abstract

Participatory community-based women’s group interventions have been successful in improving maternal and newborn survival. In rural Makwanpur, Nepal, exposure to these Participatory Learning and Action groups resulted in a thirty-percent reduction in neonatal mortality rate and significantly fewer maternal deaths. It is often theorised that participatory approaches are more likely to be sustained than top-down approaches, but this is rarely evaluated after the withdrawal of external support. We sought to understand how participatory learning and action (PLA) groups in Makwanpur fared after the supporting non-governmental organisation withdrew their support as well as factors affecting their sustainability. We used mixed methods, conducting a cross-sectional survey of 239 groups, thirty focus group discussions with group members and thirty key informant interviews within twelve–seventeen months after support was withdrawn. Eighty percent of groups were still active which suggests that PLA groups have a high chance of being sustained over time. Groups were more likely to be sustained if the group had local importance and members continued to acquire new knowledge. However, the participatory nature of the group and local embeddedness were not enough to sustain all groups. They also needed leadership capacity, a unifying activity such as a fund, and a strong belief in the value of their meeting to sustain. These key factors should be considered when seeking to enable sustainability of participatory interventions.

## Introduction

Globally, it is estimated that 289,000 women die every year of pregnancy or child-birth related complications (United Nations, 2015). Additionally, 16,000 children under five die every day, mostly from preventable causes (United Nations, 2015). Evidence-based, community-focused interventions addressing maternal and newborn health play a crucial role in preventing these deaths (Darmstadt *et al.*, 2005; Lawn *et al.*, 2014).

A review and meta-analysis of community-based women’s groups working through participatory learning and action (PLA) cycles showed that they can be effective in improving maternal and newborn survival in rural settings in low-income countries (Prost *et al.*, 2013). The meta-analysis included studies from seven trials in four countries and found that exposure to PLA groups resulted in a twenty percent reduction in neonatal mortality. Participatory interventions have been challenged for being difficult to implement at scale, and there have been calls for research to understand how these kinds of interventions can be sustained to provide ongoing health improvements (Marston *et al.*, 2013; World Health Organization, 2014).

In this paper, we focus on one of the studies included in the meta-analysis, the Mother and Infant Research Activities (MIRA) Makwanpur cluster randomised controlled trial (RCT) implemented in rural Nepal (Manandhar *et al.*, 2004). We used qualitative and quantitative data collected one year after the trial was completed to understand how sustainable the intervention was, and explore the barriers and facilitators to sustaining the group meetings and their activities. Given the impact of this and other PLA interventions on health outcomes, it is important to research issues affecting their sustainability.

### Participatory approaches to health

Since the adoption of the Alma-Ata declaration, community participation has been recognised as a key component in primary healthcare (PHC) and an important element in improving health, particularly among poor and underserved populations in developing countries (Rifkin, 1990; Rosato *et al.*, 2008). Participatory community-based approaches to health programmes have long been advocated as a cost-effective way to deliver health services. They have been considered important because of their ability to improve service utilisation as well as their greater potential for sustainability over time (Morrison *et al.*, 2005; Lassi, Haider and Bhutta, 2010). However, the concept of ‘community participation’ is an infinitely malleable concept (Cornwall, 2008), which can mean different things to different people. This has enabled different actors to use the rhetoric of participation to advance conflicting goals (Morgan, 2001). Arnstein’s ladder is often used to describe the different types of participation, or levels of engagement and the extent to which they enable citizen control over programmes (Arnstein, 1969). Laverack considers community participation as insufficient to achieve emancipation and empowerment, but recognises it as one step on a continuum towards community empowerment (Cleaver, 2001; Laverack, 2004).

‘Community participation’, is defined in the Makwanpur PLA intervention as an empowerment and capacity-building tool through which individuals or groups create a sense of solidarity, and responsibility for diagnosing and working together with their communities to solve their own health and development problems (Morgan, 2001; Howard-Grabman and Snetro, 2003; Manandhar *et al.*, 2004; Nair *et al.*, 2012). Instead of using a more traditional didactic approach focused on transmission of knowledge, community participation creates local ownership and involvement of development stakeholders that enables improved accountability, responsiveness, and embeddedness in systems. Paolo Freire emphasised that sustainable change is only possible if poor and deprived communities are engaged in dialogue, ideas, and experiences are exchanged, and they are empowered to take action to improve their own health (Freire, 1970). Yet, evidence for the sustainability of participatory community-based approaches is limited, and few organisations reflect on how successful they are in enabling sustainability (Sarriot *et al.*, 2004).

### Sustainability

The ultimate goal of development aid is sustainability (Malmqvist, 2000), yet conceptually it is difficult to define and measure. Broadly, sustainability is the continuation and success of external investment after major technical, managerial, and financial support has ceased (Bossert, 1990; LaFond, 1995; Stirman *et al.*, 2012). A definition of what constitutes ‘successful continuation’ is important, particularly given the shifting priorities and needs of populations over time. While continuation of project activities after external support withdraws is often considered a proxy for sustainability (Bossert, 1990; LaFond, 1995; Stirman *et al.*, 2012), indefinite continuation of activities is not always favourable, particularly if there is no analysis about their continued relevance or effectiveness. In this paper, we consider successful continuation of the intervention, but also consider the extent to which community and group capacity was built to ‘sustain’ or continue implementing participatory processes to improve health and development after assistance from the supporting non-governmental organisation (NGO) was withdrawn (Laverack, 2001).

### The intervention

**Figure 1.**
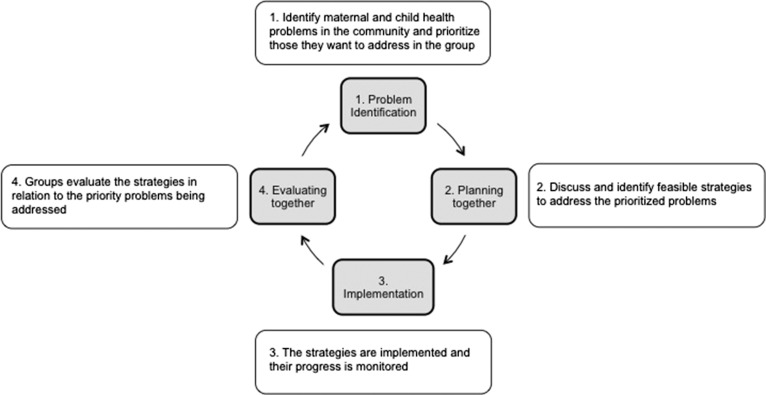
The PLA cycle

A collaboration between a Nepalese NGO, MIRA, and the Institute for Global Health, evaluated the effectiveness of women’s PLA groups on newborn mortality in rural Makwanpur District through a cluster RCT which ran from 2001 to 2003. In intervention areas, PLA groups were convened by paid local women who were not health workers or health volunteers, who had received training in facilitation skills. These facilitators led groups through twenty-four monthly meetings in a PLA cycle (Figure 1) consisting of four phases: problem identification; development of strategies to address identified problems; working together with their community on implementation; and evaluation of strategies (Morrison *et al.*, 2005). Facilitators organised meetings in coordination with the local female community health volunteer (FCHV), an unpaid community-based health worker. The facilitator used a meeting manual and picture cards to guide the group through the four phases of the PLA cycle (Figure 1). The groups were not solely intended for pregnant women, and women of various ages attended. Some men also attended groups, but they did not take a substantial role in leadership or implementation of activities. Intervention clusters experienced a thirty-percent reduction in neonatal mortality compared with control clusters, and maternal mortality was significantly lower with a maternal mortality ratio of sixty-nine deaths per 100,000 livebirths compared with 341 deaths per 100,000 livebirths (Manandhar *et al.*, 2004).

During the trial, the most common strategies implemented by the PLA groups were: (1) an emergency maternal and child health fund, (2) production and management of stretchers, (3) locally produced clean home delivery kits, and (4) awareness raising through plays and video shows (Morrison *et al.*, 2005). Women also conducted home visits to pregnant women who did not attend the groups, especially younger primigravid mothers. The PLA approach and content of the strategies is described in more detail elsewhere (Manandhar *et al.*, 2004; Morrison *et al.*, 2005, 2008, 2010). Population surveillance continued from 2003 to 2008, the intervention continued in previous intervention areas, and expanded fully to control areas in 2005. In March 2008, MIRA withdrew support and handed-over the women’s groups to FCHVs who received a four-day training to gain confidence and skills to take over the facilitation process.

## Methods

### Study location and population

Makwanpur District is located in the central region of Nepal where the hills meet the plains. It has a population of 420,477, of whom most live in rural areas and are engaged in subsistence agriculture (Central Bureau of Statistics, 2014). The largest ethnic groups are Tamang, Brahman, and Chhetri (Central Bureau of Statistics, 2014; District Coordination Committee Office, 2017). At the time of the study, Makwanpur was divided into forty-two geopolitical units of Village Development Committees (VDCs), and had a mid-range human development index rating (Government of Nepal, 2014). The district headquarters had a district hospital, which provided secondary level healthcare (Rai, Rai, Hirai, Abe and Ohno, 2001). PHC was provided at the VDC level through four Primary Health Centres, sixteen health posts and twenty-four sub-health posts (Rai *et al.*, 2001; District Coordination Committee Office, 2017). Outreach activities and immunisation clinics are still run by health workers and FCHVs (Ministry of Health and Population, 2012). The neonatal mortality was thirty-three deaths per 1000 livebirths and most deliveries occurred at home (Ministry of Health and Population, 2012).

### Sampling and data collection

We conducted a sequential mixed methods study, first collecting quantitative data that informed the qualitative study. We conducted a cross-sectional survey of one representative from 239 PLA groups (out of 240) established in the twenty-four VDCs where we conducted the cluster RCT between 2001 and 2008. One large ward contained two groups, of which only one was sampled for the survey. We recruited and trained thirteen experienced quantitative researchers, who had supervised pregnancy and delivery surveillance activities during the trial. Researchers piloted a structured questionnaire on twenty-four PLA group members (ten percent of the total sample) and made minor adjustments. Researchers approached FCHVs and those who had worked on the trial surveillance team to help locate group members in an effort to triangulate among sources and find group members in the scattered population. Group members were convenience sampled as we perceived that there was equal chance of bias in locating less active group members as active group members. FCHVs and trial surveillance team members could have perceived MIRA to be more likely to re-establish their support if the group was known to be less active. We asked group members about the nature of activities and meetings, factors influencing their activeness, and the support needed to continue the group. Options for closed questions were based on several iterations of category creation by the research team who had knowledge of the study area while interventions were not running. We collected quantitative data between April and May 2009.

The survey informed our qualitative study design, enabling us to identify and purposively sample PLA groups according to their activeness and explore the reasons for this. The senior research team, former surveillance team members and community key informants were consulted about how to define an active and a less active group. Through our questionnaire, we were able to identify which groups conducted meetings, worked on strategies and kept meeting minutes. These groups were defined as active. Less active groups were defined as not meeting regularly, and their strategies were sporadically implemented or completely dissolved. Purposive sampling allowed for the identification and selection of information-rich cases that would give a full range of experiences and depth of understanding (Patton, 2002). We purposively sampled twenty groups that were active, six groups that were less active, and four groups that had merged with other community groups (Table 1).

**Table 1:** Qualitative data collection (*n* = 60)

VDC	Active groups	Less active groups	Merged groups	Total
Ambhanjyang	3	3	1	7
Bhimphedi	2		2	4
Gogane	2	1		3
Harnamadi	4		1	5
Manthali	1	2		3
Markhu	4			4
Namtar	4			4
Total	20	6	4	30

We trained ten experienced qualitative researchers who had previously worked as PLA group supervisors during the trial. They conducted thirty focus group discussions (FGDs) with six–nine women who had either participated in the PLA groups during the trial (less active groups), or were currently participating in the PLA group (active groups). Researchers used topic guides to explore previous and current activities, reasons why women attended or did not attend groups, and the factors affecting the group’s sustainability. After each FGD, a key informant interview (KII) was conducted with a local person who did not attend the group but who was knowledgeable about the previous and current status of the PLA group (*n* = 30). Topic guides were piloted with five PLA groups, and two key informants in VDCs where study data collection was not conducted. Participants did not receive any incentives. Due to low levels of literacy, participants were asked for informed verbal consent to participate.

### Data analysis

Quantitative data were entered and analysed in Statistical Package for the Social Sciences using descriptive statistics. Qualitative data were recorded and transcribed verbatim, rendered anonymous, and translated into English. Content analysis (Krueger and Casey, 2000) of the translated transcripts was conducted by AS. After familiarisation and preliminary analysis through descriptive coding, AS and JM generated themes from the data through discussion. As the data were collected in 2009, it was important to understand our findings in the more recent context. Therefore, AS visited the study area in May–June 2016. AS discussed the findings with five researchers who were senior employees when data were collected and with JM who advised on the study implementation. In addition, AS met with a PLA group, an active FCHV and the Women’s Development Officer in Makwanpur and discussed the findings of the study in relation to the factors affecting participation in PLA groups, and the current situation of the groups. Data from these discussions were compared with data from 2009 and used to inform our results. All data were coded by AS in Nvivo qualitative analysis software, version 10.

### Ethical approval

The study received ethical approval from the Nepal Health Research Council (NHRC) and the UCL Institute of Child Health, London, UK.

## Main findings and discussion

PLA groups in Makwanpur have been effective in reducing neonatal and maternal mortality (Manandhar *et al.*, 2004). Most were also sustained one year after MIRA had withdrawn their support. Of the 239 PLA groups seventy-seven percent (*n* = 183) were active, twenty-one percent (*n = 49*) were less active, and three percent (*n* = 3) had merged with another community group. Fifty percent of groups that were active were run by an FCHV. We sought to understand the factors affecting the ability of groups to keep meeting, and explore reasons for activeness or lack thereof through qualitative data collection. Our analysis reveals that six key factors influence the sustainability of the PLA groups: local importance, acquiring knowledge, a unifying activity, the social environment, financial support, and leadership capacity.

### Local importance

PLA groups were regarded as important entities in all VDCs due to their positive effect on maternal and newborn survival. Our data collection in 2016 suggests that this is still the case. Furthermore, women highlighted that maternal and child health issues would continue to be important to women of future generations and PLA groups had taught communities about these issues and could ‘keep the community moving forward’ (active KII). Most women in active groups felt that the intervention met their health needs, was relevant to their community health situation, and enabled them to retain the information that they had learned: ‘If the programme is lost, we will only go backwards’ (active, FGD).

Some studies have questioned the ethics of expecting the most marginalised, with fewest resources, to participate in interventions without compensation (Cleaver, 2001; Ramnarain, 2014). Participation in group meetings and activities to mobilise the community to improve maternal and newborn health could arguably be considered to add to women’s work burden in this context. We found that usually only one woman per household attended the group, other female family members completed household work. For some active group members, the importance and relevance of newborn and maternal health enabled them to prioritise the group over household tasks: ‘Even if there is work at home, we leave it so that we can come to the group’ (active FGD). Research has shown that group attendance among poorer women was higher than better-off women. This was partly because of the participatory nature of the intervention, and the flexibility to organise meetings at the convenience of group members (Houweling *et al.*, 2017).

Some women across all three types of groups became disengaged with the group because they felt the problem of neonatal mortality had been solved or the information was not applicable to their personal circumstances anymore (i.e. they did not plan to have anymore children). Women expressing these views said they had little community support to restart or continue meetings, and so had ceased to meet or merged with another group. Motivated women in less active groups were often unwilling to continue investing time and energy in group activities without wider support from other women or community members. According to Houweling *et al.* (2016), community support for interventions is an important component for their success.

### Acquiring knowledge

Respondents felt that PLA groups enabled women to acquire knowledge. Almost all groups emphasised that acquiring knowledge about a topic like maternal and newborn health, which they considered important, was a strong motivator to continue attending the group. A lack of new information during meetings was also a reason for decreasing attendance. This sometimes led to groups stopping altogether or merging with another group: ‘Women think every time it is the same discussion, same topic, so they do not come’ (merged, FGD). Both active and less active groups were affected by the absence of the MIRA facilitator to lead the group and provide new information. They expressed a desire for the facilitator to lead the group again. For some inactive groups having no facilitator was the main cause for their inactiveness. Conversely, those groups that were able to introduce new topics often had a knowledgeable woman or active FCHV to lead the group. One group said that they relied on the FCHV to teach them new topics from the training she received about alcoholism, diabetes, sexual health, or family planning.

Other studies support our findings about the importance of a programme champion to the success and sustainability of community-based interventions (Shediac-Rizkallah and Bone, 1998; Johnson *et al.*, 2004; Pluye, Potvin and Denis, 2004; Scheirer, 2005; Scheirer, Hartling and Hagerman, 2008; Shigayeva and Coker, 2015). A programme champion is an individual who connects the implementing organisation and the intended recipients, and influences programme activities (Scheirer, 2005). They often have a positive influence on sustainability as they advocate for the needs of the programme, help secure resources and sustain essential elements of the innovation (Scheirer, 2005; Edwards and Roelofs, 2006).

### A unifying activity

During the PLA cycle, groups identified the cost barriers to seeking and obtaining care as a problem, and therefore established maternal and newborn health funds (Morrison *et al.*, 2005, 2008). Contribution to the fund was never compulsory for group membership. There was little concern about members ‘freeriding’ as each group developed their own policy about how money would be collected, how it would be loaned, who was entitled to it, who would manage it, and how it should be paid back. Even while MIRA was supporting groups, funds were not always used for maternal and neonatal health problems, however after MIRA withdrew, most groups expanded the scope of their funds. They were used for broader community activities or to cover the cost of medicine for health issues not related to maternal or newborn health. The fund incentivised many women to come to the group, and one woman in an active group said: ‘We are compelled to come (because we are) able to save for an emergency’ (active, FGD).

Our survey also found that the fund was important for sustainability. Out of eight options, most groups identified the fund as being the main reason they remained active. It was difficult for groups without a fund to continue meeting. Women from an inactive group said: ‘After we stopped collecting money, less women came’ (inactive, FGD). Groups without funds usually merged (*n* = 2) or became inactive (*n* = 2). Although our quantitative data shows that the fund was an important way to sustain the group, active group members felt that the fund alone was insufficient to make their group a sustained success: ‘If there is a fund but no learning, a fund will not be useful’ (active, FGD).

Studies on micro-finance programmes in Nepal indicate that the demand for savings facilities is high among women because they improve self-esteem, increase decision-making roles of women in households and generate an overall feeling of economic empowerment and autonomy (Cheston and Kuhn, 2002; Morrison *et al.*, 2008; Acharya, Bell, Simkhada, van Teijlingen, and Regmi, 2010). Our findings also indicate that the fund was valued because it gave group members a sense of independence: ‘When we save, we don’t have to be dependent on our husbands. We don’t have to beg for money’ (active, FGD). While some have warned about the dangers of funds increasing dependency instead of empowerment (Acharya *et al.*, 2010), women’s groups studies have shown that the establishment and running of the fund enabled group members to manage it themselves contributing to their own resources. This has enabled the funds to be sustained over time. Our discussions in Makwanpur in 2016 reconfirmed that funds have provided a tangible, valued benefit for group members over time.

### Social environment

The social environment of the group influenced its sustainability, with active group members discussing the importance of the groups as a supportive social gathering place where they felt comfortable and confident to share information. The sense of unity generated by this environment was considered important for the continuation of the group. ‘The main thing needed in groups is for everyone to have common goals and make decisions together’ (merged, KII).

The household environment also affected women’s ability to keep meeting. Entrenched household gender roles (Mullany, Hindin and Becker, 2005; Furuta and Salway, 2006; Acharya *et al.*, 2010) meant that some women were unable to join the PLA groups because their families were unsupportive, fearing that household duties would be neglected. Others questioned the benefits of attending, asking women: ‘What will you get from it?’ (active, KII), particularly after MIRA withdrew their support. In 2016, these questions were raised less frequently because active groups had become well embedded in the community. As groups have continued to meet, more and more community members have become aware of the group, its purpose and the benefits of attending. This has made it easier to convince family members about the value of attending, and sustain the groups over time. Other group members felt that women had become more empowered, and could now convince their family: ‘Now whatever it is, even if the family says not to go, the women decide to come. They win and they come’ (active, FGD).

### Financial support

An enabling community context is important in the sustained success of interventions and one aspect of this is access to financial support by community organisations. Every intervention cluster had at least nine PLA groups, one local administration office (the VDC) and a Women’s Development Committee (WDC). In addition, community organisations like forest user groups, and savings and credit groups operated in most of the study areas. Some local District Development budget was earmarked to be spent on women and children’s well-being. It was difficult for some groups to compete with schools, health facilities, and other initiatives for this funding, due to general scarcity of local resources, lack of appropriate contacts or lack of advocacy skills. Senior MIRA employees that we interviewed in 2016 confirmed that a large proportion of active groups still struggle to access financial support.

Sustainability frameworks often recognise these types of extra funding sources to be an important predictor of the sustainability of health programmes (Shediac-Rizkallah and Bone, 1998; Johnson *et al.*, 2004; Stirman *et al.*, 2012). Although the availability of a number of funding sources is identified as a key factor influencing sustainability, other studies summarised by Scheirer (2005) find that securing additional funding is much less a predictor of sustainability. Although women in our study felt that financial resources would have helped sustainability, they were not dependent on these resources and continued to meet and conduct their activities, even without support.

Developing a mutual relationship of support and accountability with other organisations within the community can contribute to greater sustainability (Shediac-Rizkallah and Bone, 1998; Scheirer, 2005; Shigayeva and Coker, 2015). Respondents discussed financial support, but they may have declined to mention other forms of support because they were perceived to be less valuable. It is also possible that group members did not link with other organisations because of their different focus. The literature highlights how linkages with other community organisations can facilitate sustainability (Sarriot *et al.*, 2004; Scheirer, 2005), and therefore more focus on linking organisations may help sustain PLA groups.

### Leadership capacity

Capacity to lead and manage a programme influences the sustainability of community-based interventions (Laverack, 2001; Johnson *et al.*, 2004; Sarriot *et al.*, 2004; Schell *et al.*, 2013). Our data show that the PLA groups remained active where there was a sense of ownership and they felt confident to take responsibility for the group: ‘MIRA showed us the way. They showed us the right track, and we are now confident to walk that track. Because of this, the group is still running’ (active, KII). Other active and less active PLA groups felt that MIRA withdrew before they had reached the level of confidence they needed to continue the activities on their own. They felt like ‘MIRA left in the middle’ before they fully developed their capacity (less active, FGD).

Both active and less active groups expressed a desire for the group to be led by a facilitator. Almost all of the groups requested more training rather than more financial resources in order to continue their activities. Women wanted to be taught about income generation; how to increase public awareness of the groups; and group management skills. One less active group told us: ‘If there was training for the chairperson, treasurer, and secretary on how to run the group, then we could do more’ (less active, KII). While the need for capacity and development may be real, some researchers argue that development is promoted as a technical problem to be solved by experts, and instead programmes should focus on developing confidence and empowerment in grassroots stakeholders to sustain activities and promote development (Leve, 2001; Easterly, 2015). The women in PLA groups might have possessed the skills, but lacked the confidence and community support to enact them.

All groups struggled to retain the PLA process in its entirety, which was confirmed to still be the case in 2016. According to senior MIRA employees, the groups were less systematic and did not follow all of the PLA steps. Although groups identified and discussed new problems independently, not all of them felt confident enough or felt that they had the capacity to design and implement strategies to tackle these problems. An active group felt that the PLA approach had not prepared them adequately to operate independently: ‘To grow and develop awareness, the programme should not only be a discussion group, the programme should focus on leadership and developing capacity for leadership’ (active, KII).

MIRA did enact a hand-over strategy, which involved training FCHVs for four days to build their confidence with the PLA approach and take over the facilitation process. FCHVs are local women who are responsible for promoting maternal and child health, linking communities to health facilities, and providing health education through local women’s groups (Ministry of Health and Population, 2014). They are selected by the women’s group according to criteria set out in the FCHV strategy. Studies have shown that the FCHV program ‘has very low attrition, very high motivation and very high levels of community involvement’ (Ministry of Health and Population, 2014 p4). Our findings show that fifty percent of active groups were run by FCHVs. It is unclear from our data whether this was due to the hand-over process being inadequate, lack of motivation or capacity of FCHVs, or lack of acceptability of the FCHV as a facilitator by group members. The Women’s Development Officer we interviewed in 2016 felt that there was no effective system in place to monitor and evaluate the work done by FCHVs, which made them less motivated. A recent study in India (Tripathy *et al.*, 2016) has shown that working with community-based incentivised volunteers (Accredited Social Health Activists, ASHAs) can be a successful delivery mechanism for PLA groups. Tripathy *et al.* (2016) found that the community recognition of ASHAs as agents of change was a strong incentive for them to continue the intervention.

FCHVs could be motivated to facilitate groups through financial incentives, as this has shown effective in some contexts (Singh *et al.*, 2015). Previous research in Nepal has suggested that regular salaries were regarded as a threat to the social respect that FCHVs commanded (Glenton *et al.* 2010). This social respect was a significant motivating factor for FCHVs. If they received salaries they would be seen as paid health workers motivated by money, which would negatively impact their moral status, and their social prestige could be undermined. In Nepal, FCHVs may be better motivated by improved systems of support and supervision or non-monetary incentives.

Our data suggest that FCHVs might not be the most appropriate delivery mechanism for this type of intervention, particularly given their health education focus. We found that women were motivated to attend women’s groups because facilitators were someone outside their regular social circle, who they perceived to be a trained, skilled person. FCHVs were not always held in the same regard. In Makwanpur, a phased withdrawal of support, and identification and training of group members interested in leadership positions might have enabled sustainability of the group. Where FCHVs were less-motivated or less well-respected, group members could have been mentored over time so that they could take up leadership positions or support the FCHV in running the group.

### Study limitations

It is possible that the factors affecting participation might have changed since data were collected in 2009. AS’s data collection in 2016 with a PLA group, an active FCHV and the Women’s Development Officer in Makwanpur confirmed that our findings were still relevant. Broader indicators of women’s status in Nepal, such as persisting high rates of child marriage, girl-child school drop-out, and the continuing gender discrimination evident in legal frameworks also indicate that women’s status has not made much progress since 2009 (Ministry of Women, Children and Social Welfare, 2016; Accord, 2017).

MIRA employees who implemented the trial also collected the quantitative and qualitative data for this study. This may have caused the data collection to be biased. It is also possible that difficulties experienced by the women were overstated in order to convince MIRA to re-establish their support. Validity was addressed to the greatest extent possible by triangulation through different methods of data collection: qualitative and quantitative data and between qualitative data sources (Patton, 2002). Rigour was increased by documentation and reflection on methodological decisions, negative case analysis, and consultation and discussion between AS and JM while generating themes.

## Conclusion

Considering that donor investment in developing countries has not always been effective in fostering lasting change and is criticised for creating aid dependency, it is important to consider how interventions continue after project support stops. Participatory community-based models of health service delivery and health promotion are often lauded as cost-effective and more likely to be sustained than top-down approaches. However, few projects are evaluated after withdrawal of external support. In this article, we explored how an effective intervention in rural Nepal, PLA groups, fared after the supporting NGO withdrew their support. We also explored the factors affecting their sustainability, and suggested strategies to enhance the sustainability of PLA groups.

Encouragingly, eighty percent of the 239 PLA groups remained active twelve–seventeen months after MIRA withdrew support and some groups even expanded their activities to local needs. Factors associated with successful continuation of the intervention included the presence of a unifying activity such as the fund; a strong belief in the value of their meeting; leadership capacity and a motivated facilitator. In India, incentivised community-based volunteers were a successful delivery mechanism for PLA groups (Tripathy *et al.* 2016), but in Nepal we found that without incentives, and adequate and continued support and supervision, only fifty percent of groups were run by FCHVs. Our study showed that some group members were also interested in leadership positions, and we suggest identifying one or more co-facilitators per group earlier in the PLA cycle. Co-facilitators could be mentored by the facilitator, helping to develop their confidence, leadership capacity, and increase community support for the legitimacy of their leadership, in preparation for the withdrawal of external support.

Although it is important to embed interventions within existing systems, if these systems themselves are insufficiently supported, or already working at full capacity, then interventions are less likely to be sustained. Those seeking to implement PLA interventions should consider locally appropriate incentivisation, as well as accountability, support and supervision mechanisms for facilitators. This should include a critical examination of the systems where interventions are being embedded, and exploration of alternatives. One alternative might be to merge, or link PLA groups more explicitly with other community groups, integrating this into the ‘planning together’ phase of the PLA cycle. When considering the sustainability of PLA groups, it is important to evaluate the extent to which the PLA process has been sustained, and how to sustain this through engaging new and old members in developing their own solutions to prioritised problems. We suggest further research to explore the effects of merging groups on the PLA method and on sustainability of groups.

## References

[bsy017C1] Accord (2017) Gender and Nepal’s transition from war, Accord, London, UK.

[bsy017C2] Acharya D. R., Bell J. S., Simkhada P., et al (2010) Women’s autonomy in household decision-making: a demographic study in Nepal, Reproductive Health, 7, 15. 20630107 10.1186/1742-4755-7-15 PMC2914657

[bsy017C3] Arnstein S. R. (1969) A ladder of citizen participation, Journal of the American Planning Association, 35 (4), 216–224.

[bsy017C4] Bossert T. J. (1990) Can they get along without us? Sustainability of donor-supported health projects in Central America and Africa, Social Science & Medicine, 30 (9), 1015–1023. 2336568 10.1016/0277-9536(90)90148-l

[bsy017C5] Central Bureau of Statistics (2014) National Population and Housing Census 2011: Village Development Committee Makwanpur, Central Bureau of Statistics, Kathmandu, Nepal.

[bsy017C6] Cheston S. and Kuhn L. (2002) Empowering women through microfinance, Draft, Opportunity International.

[bsy017C7] Cleaver F. (2001) Institutions, agency and the limitations of participatory approaches in development, in Cooke B. and Kothari U., eds, Participation: the new tyranny? Zed Books, London, UKb, pp. 36–55.

[bsy017C8] Cornwall A. (2008) Unpacking ‘Participation’: models, meanings and practices, Community Development Journal, 43, 269–283.

[bsy017C9] Darmstadt G. L., Bhutta Z. A., Cousens S., et al (2005) Evidence-based, cost-effective intervention: how many newborn babies can we save, Lancet, 365, 977–988. 15767001 10.1016/S0140-6736(05)71088-6

[bsy017C10] District Coordination Committee Office (2017) *Brief Introduction*, District Coordination Committee Office, accessed at http://ddcmakwanpur.gov.np/en/brief-introduction/ (3 August 2017).

[bsy017C11] Easterly W. (2015) The tyranny of experts: economists, dictators, and the forgotten rights of the poor, Basic Books, New York.

[bsy017C12] Edwards N. C. and Roelofs S. M. (2006) Sustainability: the elusive dimension of international health projects, Canadian Journal of Public Health, 97 (1), 45–49. 16512328 10.1007/BF03405214 PMC6975736

[bsy017C13] Freire P. (1970) Pedagogy of the oppressed, Penguin Books, London, UK.

[bsy017C14] Furuta M. and Salway S. (2006) Women’s position within the household as a determinant of maternal health care use in Nepal, International Family Planning Perspectives, 32 (1), 17–27. 16723298 10.1363/3201706

[bsy017C15] Glenton C., Scheel I. B., Pradhan S., et al (2010) The female community health volunteer programme in Nepal: decision makers’ perceptions of volunteerism, payment and other incentives, Social Sciene & Medicine, 70, 1920–1927. 10.1016/j.socscimed.2010.02.034 20382464

[bsy017C16] Government of Nepal (2014) Nepal Human Development Report 2014, Government of Nepal, Kathmandu, Nepal.

[bsy017C17] Houweling T. A. J., Morrison J., Alcock G., et al (2016) Reaching the poor with health interventions: programme-incidence analysis of seven randomised trials of women’s groups to reduce newborn mortality in Asia and Africa, Journal of Epidemiology and Community Health, 70 (1), 31–41. 26246540 10.1136/jech-2014-204685 PMC4717375

[bsy017C18] Houweling T. A. J., Looman C. W. N., Azad K., et al (2017) The equity impact of community women’s groups to reduce neonatal mortality: a meta-analysis of four cluster randomized trials, International Journal of Epidemiology, 1–15. doi:10.1093/ije/dyx160. 29024995 10.1093/ije/dyx160 PMC6380297

[bsy017C19] Howard-Grabman L. and Snetro G. (2003) How to mobilize communities for health and social change, Johns Hopkins Bloomberg School of Public Health, Baltimore, United States of America.

[bsy017C20] Johnson K., Hays C., Center H., et al (2004) Building capacity and sustainable prevention innovations: a sustainability planning model, Evaluation and Program Planning, 27, 135–149.

[bsy017C21] Krueger R. and Casey M. A. (2000) Focus groups: a practical guide for applied research, Sage Publications, London, UK.

[bsy017C22] LaFond A. (1995) Sustaining primary health care, Earthscan, London, UK.

[bsy017C23] Lassi Z. S., Haider B. A. and Bhutta Z. A. (2010) Community-based intervention packages for reducing maternal and neonatal morbidity and mortality and improving neonatal outcomes, Cochrane Database of Systematic Reviews, 11, 1–81. 10.1002/14651858.CD007754.pub2 21069697

[bsy017C24] Laverack G. (2001) An identification and interpretation of the organizational aspects of community empowerment, Community Development Journal, 36 (2), 134–145.

[bsy017C25] Laverack G. (2004) Health promotion practice, power and empowerment, Sage, Thousand Oaks, California.

[bsy017C26] Lawn J. E., Blencowe H., Oza S., et al (2014) Every newborn: progress, priorities, and potential beyond survival, Lancet, 384, 189–205. 24853593 10.1016/S0140-6736(14)60496-7

[bsy017C27] Leve L. G. (2001) Between Jesse Helms and Ram Bahadur: participation and empowerment in Women’s Literacy Programming in Nepal, PoLAR, 24 (1), 108–128.

[bsy017C28] Malmqvist H. (2000) Development aid, humanitarian assistance, emergency relief, monograph 46, Ministry for Foreign Aid, Sweden.

[bsy017C29] Manandhar D. S., Osrin D., Shrestha B. P., et al (2004) Effect of participatory intervention with women’s groups on birth outcomes in Nepal: cluster-randomised controlled trial, Lancet, 364, 970–979. 15364188 10.1016/S0140-6736(04)17021-9

[bsy017C30] Marston C., Renedo A., McGowan C. R., et al (2013) Effects of community participation on improving uptake of skilled care for maternal and newborn health: a systematic review, PLoS One, 8 (2), e55012. 23390509 10.1371/journal.pone.0055012 PMC3563661

[bsy017C31] Ministry of Health and Population (2012) Nepal demographic and health survey 2011, Ministry of Health and Population, Kathmandu, Nepal.

[bsy017C32] Ministry of Health and Population (2014) Female community health volunteer national survey report 2014, Ministry of Health and Population, Kathmandu, Nepal.

[bsy017C33] Ministry of Women, Children and Social Welfare (2016) National strategy for ending child marriage 2015, Ministry of Women, Children and Social Welfare, Kathmandu, Nepal.

[bsy017C34] Morgan L. M. (2001) Community participation in health: perpetual allure, persistent challenge, Health Policy and Planning, 16 (3), 221–230. 11527862 10.1093/heapol/16.3.221

[bsy017C35] Morrison J., Tamang S., Mesko N., et al (2005) Women’s health groups to improve perinatal care in rural Nepal, BMC Pregnancy and Childbirth, 5 (1), 6. 15771772 10.1186/1471-2393-5-6 PMC1079874

[bsy017C36] Morrison J., Thapa R., Sen A., et al (2008) Utilization and management of maternal and child health funds in rural Nepal, Community Development Journal, 45 (1), 75–89. 28824196 10.1093/cdj/bsn029 PMC5562271

[bsy017C37] Morrison J., Thapa R., Hartley S., et al (2010) Understanding how women’s groups improve maternal and newborn health in Makwanpur, Nepal: a qualitative study, International Health, 2, 25–35. 24037047 10.1016/j.inhe.2009.11.004 PMC5104268

[bsy017C38] Mullany B. C., Hindin M. J. and Becker S. (2005) Can women’s autonomy impede male involvement in pregnancy health in Kathmandu, Nepal? Social Science & Medicine, 61, 1993–2006. 15922498 10.1016/j.socscimed.2005.04.006

[bsy017C39] Nair N., Tripathy P., Costello A., et al (2012) Mobilizing women’s groups for improved maternal and newborn health: evidence for impact and challenges for sustainability and scale up, International Journal of Gynaecology and Obstetrics, 199, S22–S25. 10.1016/j.ijgo.2012.03.014 22883914

[bsy017C40] Patton M. Q. (2002) Qualitative Research & Evaluation Methods 3rd edition, Sage Publications, London, UK.

[bsy017C41] Pluye P., Potvin L. and Denis J. P. (2004) Making public health programs last: conceptualizing sustainability, Evaluation and Program Planning, 27, 121–133.

[bsy017C42] Prost A., Colbourn T., Seward N., et al (2013) Women’s groups practising participatory learning and action to improve maternal and newborn health in low-resource settings: a systematic and meta-analysis, Lancet, 381, 1736–1746. 23683640 10.1016/S0140-6736(13)60685-6 PMC3797417

[bsy017C43] Rai S. K., Rai G., Hirai K., et al (2001) The health system in Nepal: an introduction, Environmental Health and Preventive Medicine, 6 (1), 1–8. 21432230 10.1007/BF02897302 PMC2723647

[bsy017C44] Ramnarain S. (2014) Interrogating women’s peace work: community-based peacebuilding, gender, and savings’ co-operatives in post-conflict Nepal, Community Development Journal, 50 (4), 677–692.

[bsy017C45] Rifkin S. B. (1990) Community participation in maternal and child health family planning programmes: an analysis based on case study materials, World Health Organization, Geneva, Switzerland.

[bsy017C46] Rosato M., Laverack G., Grabman L. H., et al (2008) Community participation: lessons for maternal, newborn and child health, Lancet, 372, 962–971. 18790319 10.1016/S0140-6736(08)61406-3

[bsy017C47] Sarriot E. G., Winch P. J., Ryan L. J., et al (2004) A methodological approach and framework for sustainability assessment in NGO-implemented primary health care programs, International Journal of Health Planning and Management, 19, 23–41. 15061288 10.1002/hpm.744

[bsy017C48] Scheirer M. A. (2005) Is sustainability possible? A review and commentary on empirical studies of program sustainability, American Journal of Evaluation, 26 (3), 320–347.

[bsy017C49] Scheirer M. A., Hartling G. and Hagerman D. (2008) Defining sustainability outcomes of health programs: illustrations from an online-survey, Evaluation and Program Planning, 31, 335–346. 18835642 10.1016/j.evalprogplan.2008.08.004

[bsy017C50] Schell S. F., Luke D. A., Schooley M. W., et al (2013) Public health program capacity for sustainability: a new framework, Implementation Science, 8, 15. 23375082 10.1186/1748-5908-8-15 PMC3599102

[bsy017C51] Shediac-Rizkallah M. C. and Bone L. R. (1998) Planning for the sustainability of community-based health programs: conceptual frameworks and future directions for research, practice and policy, Health Education Research, 13 (1), 87–108. 10178339 10.1093/her/13.1.87

[bsy017C52] Shigayeva A. and Coker R. J. (2015) Communicable disease control programmes and health systems: an analytical approach to sustainability, Health Policy and Planning, 30, 368–385. 24561988 10.1093/heapol/czu005

[bsy017C53] Singh D., Negin J., Otim M., et al (2015) The effect of payment and incentives on motivation and focus of community health workers: five case studies from low- and middle-income countries, Human Resources for Health, 13, 58. 26169179 10.1186/s12960-015-0051-1 PMC4501095

[bsy017C54] Stirman S. W., Kimberly J., Cook N., et al (2012) The sustainability of new programs and innovations: a review of the empirical literature and recommendations for future research, Implementation Science, 7, 17. 22417162 10.1186/1748-5908-7-17 PMC3317864

[bsy017C55] Tripathy P., Nair N., Sinha R., et al (2016) Effect of participatory women’s groups facilitated by Accredited Social Health Activists on birth outcomes in rural eastern India: a cluster-randomised controlled trial, Lancet Global Health, 4, 119–128. 10.1016/S2214-109X(15)00287-9 26823213

[bsy017C56] United Nations (2015) The millennium development goals report 2015, Department of Economic and Social Affairs of the United Nations Secretariat, UN, New York.

[bsy017C57] World Health Organization (2014) WHO recommendation on community mobilization through facilitated participatory learning and action cycles with women’s groups for maternal and newborn health, World Health Organization, Geneva. 25165805

